# A Swirling-Flow-Enhanced Triboelectric Nanogenerator for Improved Dilute-Phase Particle Sensing

**DOI:** 10.3390/s26082284

**Published:** 2026-04-08

**Authors:** Mei Zhang, Bin Zhang, Zhaozhao Li, Jinnan Zhang, Yuhan Luo, Zhengyan Yue

**Affiliations:** 1 Marine Engineering College, Dalian Maritime University, Dalian 116026, China; 2 Power China Northwest Engineering Corporation Limited, Xi’an 710065, China

**Keywords:** swirling flow, self-powered sensing, triboelectric nanogenerator, dilute phase flow, particle enrichment, detection performance

## Abstract

Precise measurement of particle concentration in dilute gas–solid two-phase flows is challenging due to low particle loading and stochastic particle motion, which lead to weak signals and detection blind zones. This study develops a swirling-flow-enhanced triboelectric nanogenerator (SF-TENG) using active flow field regulation to enhance particle–wall interactions. Through CFD optimization of guide vane geometry, the SF-TENG achieved a nearly twenty-fold increase in short-circuit current compared to non-swirling configurations. The swirling flow exhibited a particle-size-dependent enhancement mechanism. For fine particles, the improvement was mainly attributed to an increased collision ratio. For coarse particles, it resulted from enhanced charge transfer per single impact. The swirling flow continuously improved the reliability and sensitivity of detection across all particle sizes. These findings provide valuable insights for designing highly sensitive, self-powered flow meters with minimized blind zones for gas–solid monitoring.

## 1. Introduction

In fields such as pneumatic conveying, flue gas purification, and environmental monitoring, accurately measuring the concentration of particulate matter in gas–solid two-phase flow is crucial for emission control, operational safety, and process stability [1,2,3,4,5,6,7]. However, in dilute-phase systems, the low particle loading and the stochastic nature of particle collisions make reliable signal detection challenging [8,9].

Traditional particle monitoring technologies, including laser scattering [10], optical counting [11], and filter-based gravimetric analysis [12], all have inherent limitations in such environments. Optical methods are susceptible to signal attenuation and optical window contamination [8,13,14], while weighing methods lack real-time capability and require frequent maintenance [15,16,17]. More advanced instruments, such as TEOM or β-attenuation monitors, are expensive, bulky, and reliant on external power [18,19]. These limiting factors have driven the development of self-powered sensing technologies for gas–solid flows.

Since the first triboelectric nanogenerator (TENG) was introduced in 2012 [20], it has received widespread attention for its advantages in energy harvesting and self-powered sensing, including diverse energy sources [21,22,23,24,25], strong stability [26,27,28], low manufacturing costs [22,29,30,31,32,33], high energy conversion efficiency [34,35], and flexible structural design [36,37,38]. In gas–solid two-phase flow applications, TENG has shown good potential in particle size distribution measurement [39], concentration and mass flow detection [40], wind-sand transport monitoring [41], as well as powder transport energy converters [42]. These studies highlight the dual value of TENG in energy harvesting and flow diagnostics.

Despite the progress mentioned above, the application of triboelectric sensors is still limited in dilute gas–solid flows. Owing to the low particle flux under dilute conditions, effective contact events between particles and the sensing wall become infrequent and highly stochastic, leading to very weak electrical outputs that are easily obscured by environmental noise [43,44]. This results in weak output signals, poor signal-to-noise ratios, and significant detection blind zones, especially when the particle concentration is below 5 g/m^3^.

Therefore, developing a flow field control strategy that can actively enhance particle–wall interactions is key to improving the detection accuracy of dilute-phase flow systems and reducing detection blind spots. Inspired by cyclonic separation technology, this study proposes a swirling-flow-enhanced triboelectric nanogenerator (SF-TENG). While swirl structures are widely used in engineering applications, their design objectives are fundamentally different from our sensing approach. Traditional cyclone separators use strong centrifugal forces for destructive phase separation [45,46,47]. Furthermore, existing vortex-enhanced TENGs focus on wind energy harvesting, using airflow to drive the rotation or vibration of a solid structure [48,49]. By contrast, the SF-TENG actively concentrates particles onto the sensing wall through controlled swirling, thereby enhancing the strength of the triboelectric signal and the particle detection performance.

This paper optimizes the geometric parameters of guide vanes to achieve controllable vortices of different intensities through a combination of computational fluid dynamics (CFD) simulation and experimental validation. Experimental results show that by introducing optimized vortices, the short-circuit current of the SF-TENG is increased by nearly 20 times compared to the swirl-free benchmark structure. Under ultra-dilute-phase conditions (5 g/m^3^), the signal-to-noise ratio of the SF-TENG improved from 0.8 dB for the structure without vortices to over 22 dB after optimization, and it exhibited a sensitivity as high as 2.89 nA/(g·m^−3^) for 10 μm fine particles. These significant performance improvements not only effectively reduce detection blind spots in dilute flows but also reveal the differential enhancement mechanism of vortices on particles of different sizes (enhanced collision number for fine particles and optimized single-collision charge for coarse particles). The SF-TENG provides a new approach for high-sensitivity, non-destructive monitoring of dilute gas–solid flows. 

## 2. Materials and Methods

### 2.1. Structural Design and Fabrication of the SF-TENG

**Figure 1:**
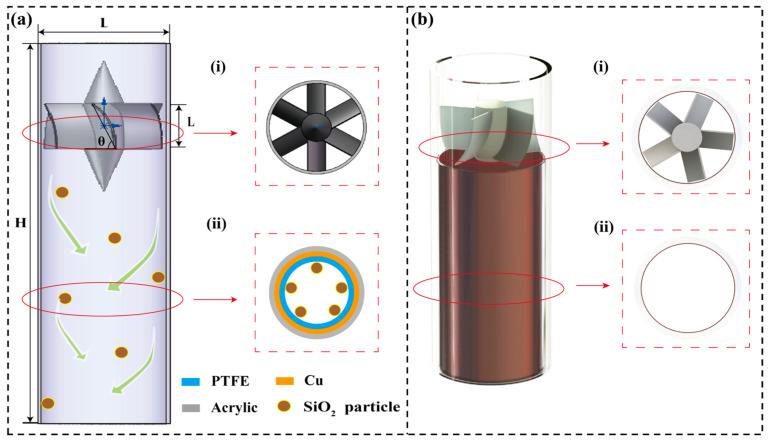
Diagram of the core structure and physical prototype of SF-TENG. (**a**) Schematic of swirling cylinder structure. (**b**) Views of the physical SF-TENG. Cross-sectional view of (**i**) guide-vane region and (**ii**) swirling flow field region.

The structure of the SF-TENG is shown in Figure 1a. The device is based on a vertically oriented cylindrical flow tube with a length of 150 mm and an inner diameter of 50 mm. As illustrated in Figure 1a, the flow channel comprises an inlet section, a guide-vane section, a swirl development section (which also serves as the active sensing region), and an outlet section.

During operation, the incoming airflow passes through the specially designed guide vanes, generating a strong swirling flow. Propelled by centrifugal forces, particles are driven toward the tube wall, where they undergo collisions and sliding interactions with the integrated triboelectric sensing unit. In this manner, the originally sparse and random particle–wall interactions under low-concentration conditions are effectively transformed into intensified and detectable electrical signal outputs.

#### 2.1.1. Guide-Vane Design and Fabrication

A set of blades uniformly distributed along the inlet circumference served as the key component for generating swirling flow [50]. The vane outlet angle, the number of vanes, and the length of vanes were adjusted to regulate swirl intensity and stability (see Table 1 for parameter ranges). All guide vanes were fabricated using polylactic acid (PLA) via fused deposition modeling (FDM) with a 3D printer (DW Maker Z6, Snapmaker, Shenzhen, China).

#### 2.1.2. Triboelectric Sensing Unit and Particle Materials

A triboelectric sensing unit was integrated onto the inner wall of the swirl development section immediately downstream of the guide vanes. A copper foil with a thickness of 100 μm was first attached to the wall as the induction electrode, followed by a polytetrafluoroethylene (PTFE) film of approximately 100 μm thickness serving as the triboelectric layer. The electrode was connected to an external data acquisition circuit via conductive wires, with conductive silver paste applied at the joints to reduce contact resistance.

Silicon dioxide (SiO_2_) particles with a density of 2.2 g/cm^3^ were selected as the test material to simulate typical industrial dust conditions [51]. The particles used in the experiment were mainly selected with two typical particle sizes: 10 μm and 1000 μm. There are two main reasons for this selection: first, they are common and typical in industrial settings; second, they effectively represent the distinct aerodynamic behaviors of fine and coarse particles, respectively, which help to reveal different mechanisms of cyclone enhancement. Due to the significant differences in the transport concentration ranges of fine particles (prone to agglomeration) and coarse particles (prone to flow) in industrial sites, this study chose to evaluate the swirling enhancement effect within their respective typical industrial concentration ranges, thereby enhancing the rationality of the experimental setup. To comprehensively evaluate the wall enrichment effect of cyclone structures on particles of different scales, the numerical simulation included a wider range of particle sizes (1 μm, 5 μm, 10 μm, 50 μm, 100 μm, and 1000 μm).

### 2.2. Experimental System and Measurement Methods

**Figure 2:**
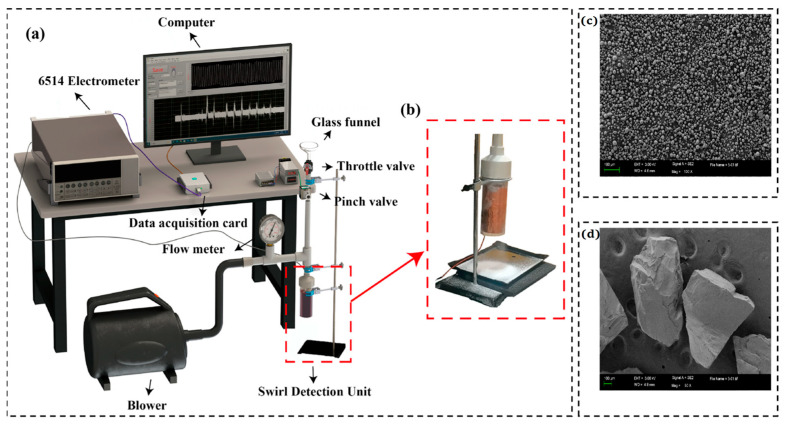
Gas–solid two-phase flow experimental platform. (**a**) Schematic diagram of the experimental system. (**b**) Enlarged view of the swirling sensing unit. SEM image of (**c**) 10 μm and (**d**) 1000 μm SiO_2_ particles.

The overall schematic diagram of the gas–solid two-phase flow experimental platform is shown in Figure 2a. Figure 2b shows an enlarged view of the swirling-enhanced sensing unit. The morphology of two representative particle sizes used in the experiment was characterized by scanning electron microscopy (SEM), as shown in Figure 2c,d. A blower and a flowmeter are used together to provide stable and adjustable inlet airflow. Particles are fed through a funnel and throttle valve. A timer relay controls a WKO4 pinch valve (Hangzhou Wokun Technology Co., Ltd., Hangzhou, China) that squeeze and releases a 6 mm rubber tube. This produced a pulsed dilute-phase flow (1 s open/4 s closed), generating periodic signals for analysis.

All experiments were conducted in a constant temperature (25 ± 2 °C) and constant humidity (relative humidity 45 ± 2%) environment. The pipelines, elbows, and other components used in the experimental setup were all made of the same material, polyvinyl chloride (PVC), to better simulate real-world application conditions. PVC is frequently employed in pneumatic conveying pipelines due to its excellent chemical stability, mechanical strength, electrical insulation, and cost-effectiveness [52]. In all comparative experiments, experimental conditions such as the length and diameter of the PVC pipes and the airflow purging time were kept strictly consistent to ensure the consistency of the initial pre-charged amount in each set of experiments. In the experiment, a Keithley 6514 electrometer (Keithley Instruments, Cleveland, OH, USA) was used to measure the short-circuit current, transferred charge, and open-circuit voltage of the SF-TENG. All measurements were repeated at least three times, and error bars represent standard deviation.

### 2.3. Numerical Simulation Framework

#### 2.3.1. Governing Equations and Model Selection

ANSYS Fluent 2020 R2 was used to simulate the three-dimensional transient gas-particle dynamics. High streamline curvature and anisotropic turbulence are inherent characteristics of swirling flows. To accurately resolve the turbulence stress components in the swirling flow, the Reynolds Stress Model (RSM) was utilized [53]. The governing equations are expressed as follows:

(1) $$\frac{\partial u_{i}}{\partial x_{i}}=0$$

(2) $$\frac{\partial }{\partial t}\left(\rho u_{i}\right)+\frac{\partial }{\partial x_{j}}\left(\rho {u_{i}u}_{j}\right)=-\frac{\partial p}{\partial x_{i}}+\frac{\partial }{\partial x_{j}}\left[\mu \left(\frac{\partial u_{i}}{\partial x_{j}}+\frac{\partial u_{j}}{\partial x_{i}}\right)-\rho \bar{u_{i}^{’}u_{j}^{’}}\right]$$

To resolve the discrete particle trajectories, the Discrete Phase Model (DPM) was integrated within a Lagrangian framework. The motion of a particle is governed by the force balance equation:

(3) $$\frac{du_{p}}{dt}=\frac{\left(u-u_{p}\right)}{\tau_{r}}+\frac{g(\rho_{p}-\rho )}{\rho_{p}}$$

where u is the gas velocity; $u_{p}$ is the particle velocity; ρ is the gas density; $\rho_{p}$ is the particle density; $\tau_{r}$ is the particle relaxation time; g is the acceleration of gravity.

Under the operating conditions established in this experiment, the particle mass concentration at the inlet of the SF-TENG is from 4 to 1400 g/m^3^, which corresponds to a particle volume fraction of around 1.8 × 10^−6^ to 6.3 × 10^−4^. The range satisfies the criteria of a dilute phase, in which the common dilute-phase criterion is that the volume fraction of particles should be less than 10^−3^. In the flow state of a dilute phase, inter-particle collisions can be neglected, and the particles have little interaction with the carrier fluid. Thus, it is reasonable to use a one-way coupled DPM.

#### 2.3.2. Boundary Conditions and Mesh Strategy

**Figure 3:**
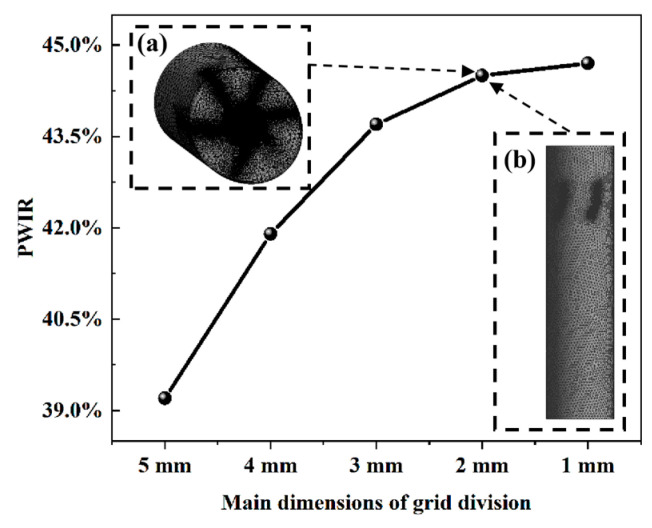
Mesh generation and independence verification for CFD simulation. (**a**) 2 mm mesh distribution of the SF-TENG (global view). (**b**) Local refinement at the guide vane region.

In the Fluent simulations, the wall boundary condition was set as a trap for particles. The vane surfaces were set as reflect, and all other boundaries as escape. The mesh independence test is shown in Figure 3. Compared with the 1 mm mesh size, the PWIR difference under the 2 mm mesh is less than 1%, while the computational cost is significantly reduced. Ultimately, a polyhedral mesh with a global size of 2 mm (about 8.4 million cells) was selected to balance computational accuracy and efficiency. Figure 3a shows the mesh distribution of SF-TENG under the 2 mm mesh, with a focus on the local refinement of key areas (Figure 3b).

#### 2.3.3. Core Evaluation Metric

To assess the particle enrichment effect induced by the swirling flow, the Particle–Wall Impact Rate (PWIR) is used as the core evaluation metric. PWIR is defined as the ratio of the number of particles contacting the wall to the total number of particles at the inlet:

(4) $$PWIR\;=\;\frac{N_{\mathrm{trap}}}{N_{\mathrm{inlet}}}\;\times \;100\%$$

Here, N_inlet_ represents the total number of particles entering through the SF-TENG inlet, and N_trap_ denotes the number of particles trapped by the wall under specific structural parameters.

In CFD simulations, this value is directly computed within the DPM framework by tracking the fate of each individual particle trajectory. But it is difficult to directly count the number of particle–wall collisions through experiments, this study uses a mass weighing method to experimentally approximate PWIR. This method is implemented in separate parallel experiments under the same operating conditions, which are conducted independently of the electrical signal measurements. A thin layer of double-sided tape is evenly applied to the inner wall of the swirl tube to capture particles that collide with the wall. Given that the SiO_2_ particles used in this study show a relatively narrow size distribution and have a constant density, the particles have roughly equal mass. Therefore, the ratio of the mass of particles deposited on the wall (M_wall_) to the total mass of injected particles (M_in_) can serve as a reasonable approximation for the PWIR defined in Equation (4).

## 3. Results and Discussion

### 3.1. Sensing Principle and Theoretical Model

**Figure 4:**
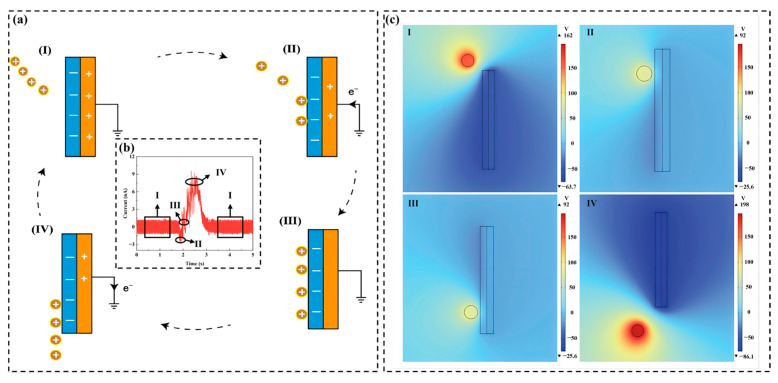
Working principle of the SF-TENG. (**a**) Schematic of charge generation through particle–wall contact in swirling flow. (**b**) Alternating current signal waveform. (**c**) Spatial potential distribution simulated by COMSOL.

TENG generates opposite charges at the interface through the periodic contact and separation of two materials with different electron affinities, forming an alternating current output. Figure 4 illustrates the working principle of the SF-TENG. Its core mechanism combines contact electrification and electrostatic induction (Figure 4a). During transport, SiO_2_ particles acquire a positive pre-charge through friction with the PVC pipe. When they enter the induction region, centrifugal force pushes them toward the sensing wall, where they approach, contact, and leave the PTFE layer. This process causes periodic changes in the local electric field, driving alternating electron transfer between the copper electrode and the ground [41]. Through pulse feeding, microscopic charge transfer is converted into a synchronized macroscopic alternating current (AC) signal (Figure 4b). COMSOL Multiphysics 6.2 simulations further reveal the spatial potential distribution in this process (Figure 4c), supporting that particle–wall interactions are effectively transformed into detectable electrical signals.

The physical foundation of the SF-TENG for solid-phase concentration monitoring in dilute gas–solid flows lies in the statistical correlation between the macroscopic output signal and the frequency of microscopic particle–wall collision events. During the dynamic process of contact electrification and electrostatic induction, the total transferred charge $Q_{total}$ can be regarded as the macroscopic superposition of numerous discrete collision events. If $q_{0}$ is defined as the average charge transferred during a single effective collision between a particle and the triboelectric layer, the total charge transferred per unit time can be expressed as:

(5) $$Q=N\cdot q_{0}$$

Here, N represents the number of effective collisions that occur during a single particle flow collision process. To establish a quantitative link between the electrical signal and the mass concentration C, it is necessary to analyze the source of the collision number N. The total mass flow rate of particles entering the SF-TENG is given by C⋅v⋅A, where v is the gas velocity and A is the cross-sectional area of the tube. Given the average mass of a single particle m_p_, the total number of particles entering the system per unit time is $(C\cdot v\cdot A)/m_{p}$. By introducing the effective collision probability p (the PWIR defined previously) to characterize the influence of flow field regulation on particle motion, the number of particles actually making effective contact with the wall per unit time, N, is expressed as:

(6) $$N=p\cdot \frac{C\cdot v\cdot A}{m_{p}}$$

By substituting Equation (6) into Equation (5), the mapping equation between the SF-TENG output signal and the particle concentration C is obtained:

(7) $$Q_{total}=(\frac{q_{0}\cdot v\cdot A\cdot p}{m_{p}})\cdot C$$

Since the short-circuit current $I_{sc}$ is physically defined as the rate of charge transfer over time

(8) $$I_{sc}=\frac{dQ}{dt}$$

a significant linear proportional relationship exists between the output current and the particle concentration under steady-state operating conditions:

(9) $$I_{sc}\propto (\frac{q_{0}\cdot v\cdot A\cdot p}{m_{p}})\cdot C$$

To further measure the sensor gain, the response sensitivity S is defined as the derivative of the short-circuit current with respect to particle concentration:

(10) $$S=\frac{dI_{sc}}{dC}\propto \frac{q_{0}\cdot v\cdot A\cdot p}{m_{p}}$$

This theoretical framework (Equations (5)–(10)) establishes the fundamental sensing principle of SF-TENG. Equation (10) indicates that for a given particle size $m_{p}$ and flow velocity v, the sensor gain is directly modulated by the swirling-enhanced collision probability p and the charge transfer efficiency $q_{0}$.

### 3.2. Enhancement Mechanism of Swirl Structure on TENG

**Figure 5:**
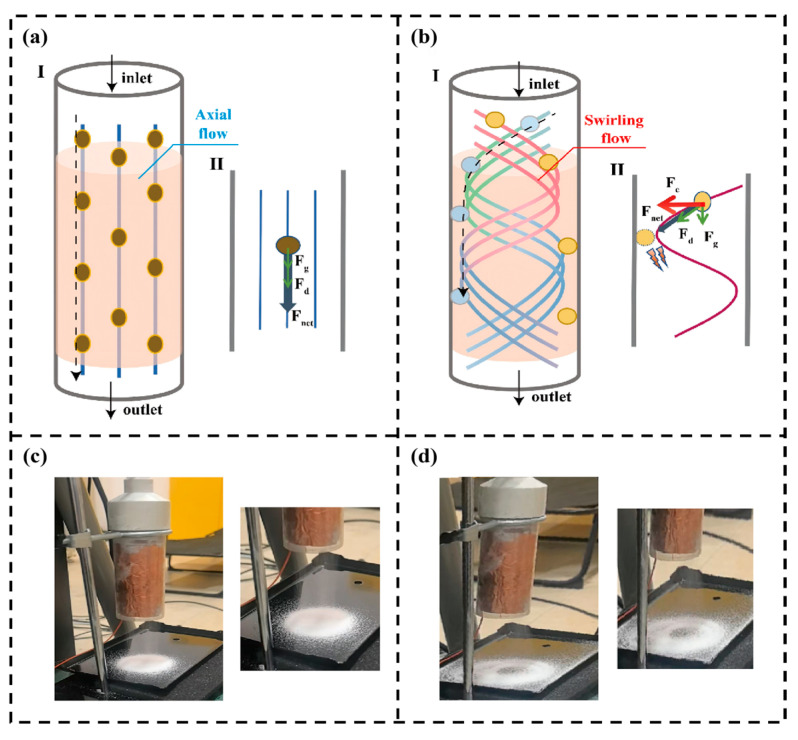
Comparison of particle behavior in axial and swirling flow fields. (I) Particle motion (II) and force analysis in (**a**) axial flow and (**b**) swirling flow. Outlet accumulation pattern under (**c**) axial flow and (**d**) swirling flow.

To enhance these key parameters, especially the effective collision probability p (PWIR), this section introduces a swirl control strategy to maximize particle–wall interactions through centrifugal enrichment. Figure 5 compares the motion and forces on particles in axial flow and swirling flow. In a conventional axial flow channel (Figure 5a), particles are mainly affected by aerodynamic drag force F_d_ and gravity F_g_. Since there is no radial force pushing them toward the wall, most particles exit directly through the outlet, with only a few colliding with the wall due to random disturbances or initial positions close to the wall. Therefore, the probability of particles contacting the annular electrode region is very low. This severely limits the generation of effective signals, especially under low particle flux conditions.

In the swirling field (Figure 5b), particles are subjected to a centrifugal force F_c_, which drives them toward the wall. Although the aerodynamic drag F_d_ tends to move particles along the streamlines, the centrifugal force overcomes radial resistance. Driven by the net force F_net_, the particles acquire radial velocity, cross the streamlines, and migrate toward the wall [54]. After the particles pass through the swirling detection unit, the accumulation pattern of the particles can be observed on the plate at the outlet of the swirl tube. Under axial flow (Figure 5c), the particles are discharged directly from the outlet along the streamlines and accumulate evenly on the plate. Under swirling flow conditions (Figure 5d), the particles are pushed toward the wall and spiral along the wall until discharged, forming a distinct ring-shaped accumulation pattern on the plate. The two different accumulation patterns reflect the different motion behaviors of the particles under axial flow and swirling flow.

Although the experimental accumulation pattern (Figure 5c,d) provides macroscopic evidence of particle migration toward the wall under swirling flow, the interaction between the gas phase and solid particles still requires further study. CFD simulations of the SF-TENG were conducted to analyze the flow field and gas-particle coupling. The discrete phase was set as 100 μm SiO_2_ particles with an inlet velocity of 5 m/s. This velocity was below the typical suspension velocity for 100 μm particles in horizontal transport, representing a challenging low-speed deposition condition common in industrial applications. This highlights the importance of using a swirl to actively control particle motion.

**Figure 6:**
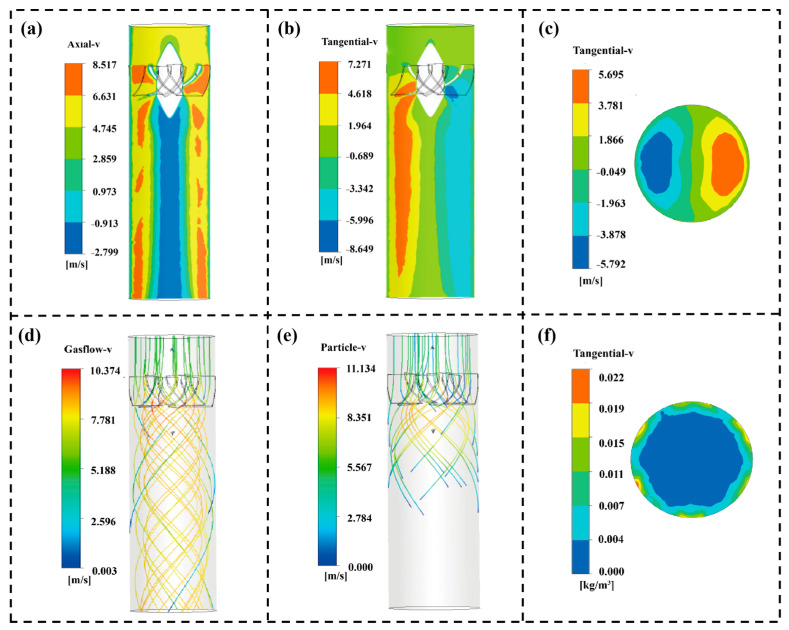
Simulation contours of flow field structure and particle motion. (**a**) Axial velocity contour (XY plane). (**b**) Tangential velocity contour (XY plane). (**c**) Tangential velocity contour (Z plane). (**d**) Streamline of swirling airflow. (**e**) Particle trajectory toward the wall. (**f**) Particle concentration contour (Z plane).

Figure 6a shows the axial velocity contour. The near-wall region appears in red, indicating high axial velocity, while the central core is blue, representing a low-speed zone. This distribution is characteristic of swirling flows, where the flow maintains high momentum near the wall. Figure 6b,c shows the tangential velocity fields on the XY plane and Z plane. In Figure 6b, one side of the section shows positive tangential velocity (red), reaching up to 7.271 m/s, while the other side shows negative tangential velocity (blue), down to −8.649 m/s. A similar pattern is observed in Figure 6c, with two opposing high-velocity regions. These symmetrical, high-magnitude tangential velocity areas indicate that the guide vanes effectively convert axial momentum into tangential momentum, generating a strong swirling flow.

The streamline in Figure 6d shows that the axial inflow quickly evolves into a tightly wrapped spiral path after passing through the guide vanes. Driven by centrifugal force, the particles deviate from the main air flow, move steadily towards the wall, and finally form a stable near-wall spiral sliding movement, as shown in Figure 6e. The cross-sectional solid phase concentration distribution in Figure 6f confirms the resulting particle enrichment. A high-concentration particle enrichment band forms in the near-wall region, and there are negligible particles in the central core [55]. The swirling structure drives the particles to create this spatial redistribution and effectively enhance the PWIR. This provides a dynamic basis for the subsequent improvement of electrical signals and detection performance.

### 3.3. Optimization of Guide-Vane Structures and Selection of Representative Configurations

**Figure 7:**
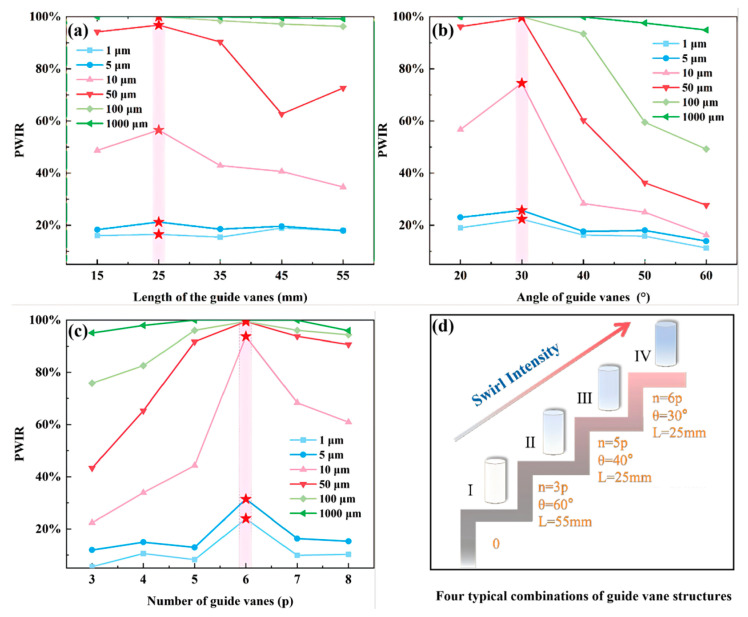
Effect of different guide vane parameters on PWIR of typical particle sizes (Range: 1 μm–1000 μm). (**a**) Length of guide vanes; (**b**) Outlet angle of guide vanes; (**c**) Number of guide vanes. The red star indicates the maximum value under the parameters of this guide vane. (**d**) Simplified schematic diagrams of four SF-TENGs with typical blade structure combinations.

According to the theoretical framework established in Section 3.1, the effective collision probability PWIR is the main factor in enhancing the signal-to-noise ratio and sensitivity in dilute-phase sensing. A parametric sweep of the three guide vane parameters (length, outlet angle, and number) reveals a non-monotonic relationship between PWIR and each parameter over a wide particle size range (1–1000 μm). Figure 7a shows the variation in PWIR for particles with changes in the length of guide vanes. Taking 10 μm particles as an example, as the vane length increases from 15 mm to 25 mm, PWIR initially increases, and then PWIR decreases with continuously increasing vane length. The length of the guide vane reflects the balance between the swirling development distance and the accumulation of flow loss [56]. A length of 15–25 mm allows the swirling motion to fully develop, whereas an extended length of 55 mm introduces excessive frictional dissipation, attenuating the swirl and reducing the PWIR. 

Figure 7b shows the PWIR of particles with the change in the guide vane outlet angle. The PWIR initially increases and then decreases with increasing outlet angle, reaching its peak at 30°. The outlet angle determines the conversion of axial momentum to tangent momentum. A small angle (20°) will give an insufficient swirl, limiting the migration of particles to the wall. However, excessive angles (>40°) will induce strong flow rotation, flow separation and energy loss, which together weaken the swirling [50,56]. Simulation results show that a 30° outlet angle achieves the best balance between swirl intensity and flow stability, allowing particles of different sizes to achieve the highest PWIR.

Figure 7c further reveals the influence of the number of guiding vanes. As the number of guide vanes increased from 3 to 6, the uniformity of the swirling flow and centrifugal force continuity significantly improved, resulting in a great increase in PWIR. When the vane number is further increased to 8, the flow channel becomes too narrow, and introduces the blockage effect, reducing the PWIR.

Taken together, Figure 7a–c show the PWIR variation trends for particles of different sizes as guide vane parameters change. They reveal that the particle PWIR response to changes in the vane structural parameters has a clear size dependence. Particles with smaller diameters (1–5 μm) have very weak inertia and a strong tendency to follow the flow, so their particle–wall collision rates remain at a low level, with very little variation in the values. Particles with diameters between 10 and 100 μm exhibit significant fluctuations in particle–wall collision rates in response to changes in guide vane parameters. For larger particles with diameters greater than 100 μm, their PWIR is almost 100% due to their dominant inertial effects, and these rates show little change even with variations in the geometry of the guide vanes.

According to the response trends observed in Figure 7a–c, four representative swirling configurations were selected based on parametric analysis. Configuration I was a straight circular tube without guide vanes, serving as the baseline condition without swirl. Configuration II (60°, 3 vanes, 55 mm) was selected from the edge area of the parameter space. Although this configuration can generate swirling flow, the swirling intensity is limited due to frictional dissipation and non-uniform flow fields. Configuration III (40°, 5 vanes, 25 mm) was selected near the inflection point of the performance curve, representing an intermediate state with a noticeable increase in swirl intensity. Configuration IV (30°, 6 vanes, 25 mm) was selected at the integrated optimization point and identified as the optimal design, capable of generating the strongest swirl. These configurations provide a physical framework for subsequent performance analysis.

### 3.4. Enhancement of Particle Detection Performance by the SF-TENG

The theoretical framework in Section 3.1 reveals two key factors that affect the sensor’s electrical signals and sensitivity. These factors are the effective collision probability p and the charge transfer per collision q_0_. This section experimentally verifies how swirling flow regulation enhances these parameters through two particle-size-dependent pathways.

#### 3.4.1. Enhancement of Detection Reliability and Signal-to-Noise Ratio for Fine Particles

**Figure 8:**
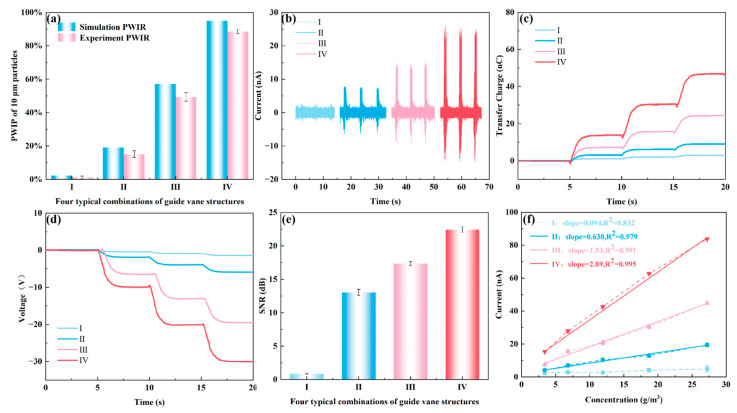
Electrical signal behavior and sensing performance of 10 μm particles under four different swirl configurations. (**a**) Simulation PWIR and experimental PWIR; (**b**) Short-circuit current; (**c**) Transferred charge; (**d**) Open-circuit voltage; (**e**) Signal-to-noise ratio (SNR); (**f**) Response of current to concentration.

For 10 μm fine particles, their inertia is small, and they easily follow the airflow closely [57]. Therefore, in the absence of a swirl, it is difficult for them to spontaneously contact the wall. Figure 8a compares the simulated PWIR and experimentally measured PWIR of 10 μm particles under four different swirling configurations. Under configuration I with no swirl, both the simulated and experimental PWIR values are low (around 2%). Most particles failed to contact the sensing wall and could not effectively generate electrical signals. With increasing swirl intensity from Configuration I to IV, the PWIR increases significantly. Under the optimal configuration IV, the experimental PWIR exceeds 88% and the simulated value is 98.6%, indicating that the optimized SF-TENG can drive the vast majority of 10 μm particles to the sensing wall using a swirling flow, significantly improving the wall contact ratio of fine particles. The experimental PWIR values are consistently slightly lower than the simulated values, with a relative deviation within 20%. This deviation may stem from practical factors, such as imperfect adhesion of double-sided tape, differences between the actual particle size distribution and the idealized CFD model, as well as inherent turbulence fluctuations and experimental uncertainties [58]. However, with the optimization of the swirl guide vane parameters, they all maintain the same monotonic increasing trend.

Figure 8b–d respectively show the short-circuit current, transferred charge, and open-circuit voltage of SF-TENG with four different swirl configurations under a particle feed rate of 3 g/min and an airflow velocity of 5 m/s. The mass concentration of gas–solid flow can be calculated as follows:

(11) $$C=\frac{m}{t\cdot v\cdot A}$$

where C is the mass concentration of particles (g/m^3^), m is the mass of inlet particles (g), t is the valve release time (s), v is flow velocity (m/s), A represents the cross-sectional area (m^2^) of the SF-TENG. When t = 1 s, v = 5 m/s and $A=\pi \cdot 0.025^{2}$ m^2^, the mass concentration is approximately 5 g/m^3^. At the same time, we achieved different feeding rates by changing the valve opening and conducted fitting calibration tests for other concentrations at an airflow speed of 5 m/s.

Compared with the weak and randomly distributed electrical signals observed in configuration I without swirl, the electrical signals of the other three configurations increase with swirl intensity. Under the optimal configuration IV, the short-circuit current signal of the SF-TENG exhibits a pulse response synchronized with the feeding cycle, and it is nearly 20 times higher than in the no-swirl configuration I. As shown in the enlarged inset in Figure 8b, each macroscopic current peak is essentially a superposition of numerous high-frequency micro-signals. For 10 μm fine particles, the frequent random micro-collisions between particles and the wall, combined with the typical powder agglomeration effect during transport, result in multiple discrete micro-currents superimposed within a single feeding pulse.

Figure 8e compares the signal-to-noise ratio (SNR) of the short-circuit current for the four different swirl configurations. In no-swirl configuration I, the SNR is only 0.8 dB, where the electrical signal is nearly completely drowned out by background noise, representing a typical detection blind zone at approximately 5 g/m^3^. As the swirl intensity increases, the SNR gradually improves. The SNR exceeds 22 dB under the same particle concentration in Configuration IV, indicating that the electrical signal is now clearly distinguishable from the background noise. As shown in Figure 8f, with the optimization of the swirling structure of the SF-TENG, the response of current to concentration exhibits a big improvement. The response slope rises from almost no response in Configuration I to 2.89 nA/(g·m^−3^) in Configuration IV. The experimentally measured linear slope (2.89 nA/(g·m^−3^) corresponds to the sensitivity S in the theoretical Formula (10). This proves that the theoretical model established in 3.1 can predict the sensor behavior. The current shows a stable linear relationship with particle concentration, and a high correlation coefficient (R^2^ > 0.979) supports this linear relationship. This suggests that the swirling structure can enhance the detection proportion of fine particles under dilute flow conditions. The centrifugal effect overcomes the particle’s tendency to follow the airflow, directing particles toward the near-wall region and increasing the proportion of effective contact with the triboelectric interface.

#### 3.4.2. Enhancement of Detection Sensitivity and Response Capability for Coarse Particles

**Figure 9:**
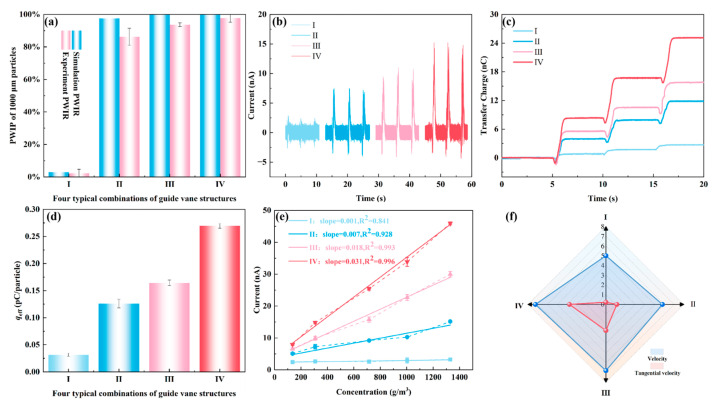
Electrical signal behavior and sensing performance of 1000 μm particles under four different swirl configurations. (**a**) Simulation PWIR and experimental PWIR; (**b**) Short-circuit current; (**c**) Transferred charge; (**d**) Average transferred charge per particle *q*_eff_; (**e**) Response of current to concentration; (**f**) Radar chart of near-wall average velocity and tangential velocity.

Unlike the trend of PWIR for 10 μm fine particles significantly increasing with the swirl intensity, for 1000 μm coarse particles, due to their larger inertia, they are prone to collide with the wall even under relatively weak swirl conditions. Figure 9a compares the simulated and experimental PWIR for 1000 μm particles. Although the CFD simulation predicts a PWIR close to 100% for Configurations II to IV, the experimental PWIR does not reach such high saturation values. This slight deviation is mainly due to the rebound effect of particles in the experiment. When large particles collide, their kinetic energy is relatively high and may overcome the local adhesion work of the collection tape, causing some particles to bounce back without being successfully captured [59,60]. In numerical simulations, upon contacting the wall, particles are considered captured and their motion is terminated, without accounting for the wall rebound effect. The experimental PWIR for Configurations II to IV increases from 86.2% to 97.8%, indicating that the number of particles contacting the wall at the same concentration does not differ much. This slight increase in PWIR cannot mathematically explain the more than twofold increase observed in the short-circuit current (Figure 9b) and transferred charge (Figure 9c).

To quantitatively assess the variation in the average charge transferred during a single effective collision between a particle and the triboelectric layer $q_{0}$ defined in the 3.1 theoretical model, this study introduces the experimental parameter $q_{eff}$, which is defined as follows:

(12) $$q_{eff}=\frac{Q_{total}}{N_{total}\times {PWIR}_{exp}}$$

Here $\;Q_{total}$ is the total transferred charge measured by a Keithley 6514 electrometer during the experiment; $N_{total}$ is the total number of injected particles, obtained by dividing the total injected mass by the single-particle mass. ${PWIR}_{exp}$ is the PWIR measured experimentally by the weighing method. $q_{eff}$ reflects the charge transfer efficiency during a particle flow collision process. Thus, $q_{eff}$ serves as an experimental estimate of $q_{0}$, the average charge transferred per single effective collision.

As shown in Figure 9d, with the increase in swirl intensity, $q_{eff}$ increases significantly, reaching its maximum value under the optimal Configuration IV. This trend is highly consistent with the theoretical model established in Section 3.1 (Equation (10)), validating the model’s effective description of the swirling enhancement mechanism. It indicates that the output enhancement mainly originates from the optimization of the collision process, and the increase in swirl intensity enhances the charge transfer in particle–wall interactions. The slope of the current–concentration response curves in Figure 9e increases substantially with swirl intensity, with the sensor sensitivity improving from 0.001 nA/(g·m^−3^) under the non-swirling Configuration I to 0.031 nA/(g·m^−3^) under the optimal Configuration IV. At the same dilute-phase concentration, the optimized SF-TENG achieved a 30-fold increase in sensitivity compared to the non-swirl state, demonstrating a significantly enhanced capability to capture small concentration variations.

To reveal the hydrodynamic origin of the charge transfer of *q*_eff_ enhanced by swirling flow, we selected eight circumferentially distributed velocity monitoring points in the near-wall region of the SF-TENG and conducted a comparative analysis of the near-wall average velocity and tangential velocity under different configurations. As shown in Figure 9f, the near-wall average velocity changes little with increasing swirl intensity, while the tangential velocity component increases significantly. For coarse particles with larger inertia, a higher tangential velocity may extend the sliding contact distance of the particles along the wall, thereby increasing the amount of charge transferred in a single collision, achieving an enhancement of the electrical signal under the same inlet solid-phase concentration. This mechanism helps to improve the response gain of coarse particles, making the electrical signal easier to detect under low concentration conditions, thereby effectively lowering the detection limit.

Although the two particle sizes were fed using the same mechanism (6 mm pinch valve, same operating frequency), their final mass concentration ranges differed significantly (approximately 30 g/m^3^ for 10 μm particles versus 1400 g/m^3^ for 1000 μm particles). This difference arises from their distinct rheological properties. 1000 μm coarse particles are dominated by gravity, have good flowability, and produce significantly higher mass flow. 10 μm fine particles have strong cohesion (such as van der Waals forces and electrostatic forces) and low bulk density, so they exhibit restricted viscous flow when passing through a 6 mm aperture.

Despite the higher mass concentration of coarse particles, their current output is lower. This can be attributed to particle number density and specific surface area. The number density of 10 μm particles is roughly six orders of magnitude higher than that of 1000 μm particles, and their total surface area is about 100 times larger, providing more contact sites and charge transfer per unit time. Although the sensing performance values differ greatly between the two particle sizes, the swirling flow significantly enhances detection performance for both. Moreover, the SF-TENG maintains high linearity (R^2^ > 0.99) at a fixed particle size, ensuring reliable quantitative sensing in dilute-phase flow.

Table 2 provides a comprehensive comparison between the proposed SF-TENG and other TENG sensors used for gas–solid flow detection. Due to differences in particle materials and size distributions, direct numerical comparisons across studies should be interpreted with caution, but the table highlights a key shift. Traditional axial-flow TENGs rely on the passive and random diffusion of particles, resulting in severe detection blind zones under dilute conditions [40,41]. In contrast, the SF-TENG achieves non-destructive active particle enrichment. The optimized configuration demonstrates sensitivity of 2.89 nA/(g·m^−3^) (for 10 μm particles) and a signal-to-noise ratio of >22 dB even at ultra-dilute concentrations as low as approximately 5 g/m^3^. This effectively reduces the detection blind zone common in conventional straight tubes under dilute conditions and offers a new approach for high-sensitivity, non-destructive monitoring of dilute gas–solid flows.

### 3.5. Long-Term Stability and Mechanical Durability

To evaluate the long-term reliability of the SF-TENG in dilute-phase flow industrial monitoring, a stability assessment was conducted. It is important to note that this assessment spanned a chronological period of six months, during which the sensor was subjected to intermittent testing. Over this six-month period, the PTFE sensing layer actively operated and continuously endured swirling high-speed particle impacts for a cumulative duration of approximately 45,000 s. The test was performed under the optimal IV configuration, comparing the output signals at the beginning and at the end of the experiment.

**Figure 10:**
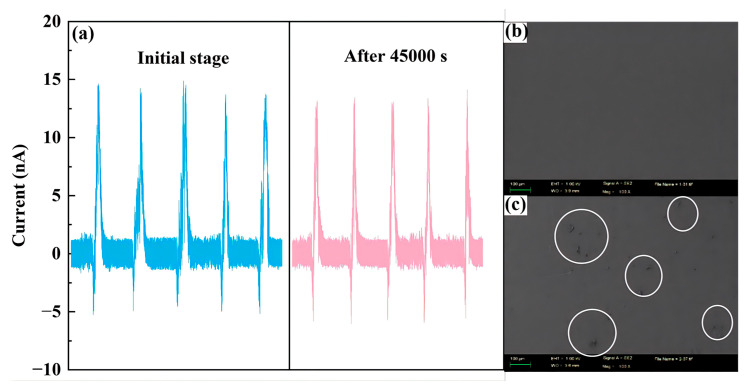
Preliminary durability assessment of SF-TENG. (**a**) Comparison of output current waveforms on initial stage and after 45,000 s under Configuration IV. SEM images of PTFE surface before (**b**) and after (**c**) 45,000 s of continuous operation.

As shown in Figure 10a, the short-circuit current after 45,000 s decays very little, less than 5%. This indicates that even after long-term exposure to particle erosion in a swirling flow, the frictional electrification efficiency of PTFE remains intact. To further verify its mechanical durability, we characterized the surface morphology of the PTFE using a scanning electron microscope. As shown in Figure 10b,c, the surface of the PTFE film remains basically intact after six months, with only minor localized micro-scratches and no delamination or peeling. These results indicate that the PTFE sensor unit has good wear resistance and can withstand long-term use in industrial environments.

## 4. Conclusions

In this study, an SF-TENG was developed as an effective strategy to mitigate detection blind zones and improve sensitivity in dilute gas–solid flow monitoring. Through a combination of experimental measurements and CFD simulations, the geometric parameters of the guide vanes were systematically optimized to control the transition from non-swirling to strongly swirling regimes. The optimized SF-TENG achieved nearly a twenty-fold increase in short-circuit current under dilute flow conditions. The proposed SF-TENG enhances the detection performance of different-sized particles through two complementary mechanisms. For fine particles, the strong swirl significantly increased PWIR, thereby enhancing the signal-to-noise ratio (SNR) and overall detection stability. For large particles (1000 μm in this study), the high tangential velocity generated by the swirl enhanced effective charge transfer during particle–wall interactions, thereby improving the sensor’s response at low concentrations. This study demonstrates that SF-TENG, thanks to active flow field control, has a significant detection advantage over traditional passive diffusion sensors at ultra-low concentrations (<5 g/m^3^), providing a universal solution to the blind zone problem. These findings provide valuable design insights for developing high-performance, self-powered sensing systems for industrial emission monitoring and pneumatic conveying applications.

## Figures and Tables

**Table 1:** Design scheme for structural parameters of the swirling guide vanes.

Parameter Name/Unit	Parameter Symbol	Variable Range
Guide vane length/mm	L	15, 25, 35, 45, 55
Guide vane outlet angle/°	θ	20, 30, 40, 50, 60
Guide vanes number/p	N	3, 4, 5, 6, 7, 8

**Table 2:** Related research comparison.

Data Source	Target Application	Flow Field Type	Core Mechanism	Measurable Concentration Range	Sensitivity	SNR/Limit of Detection
Wang et al., 2021 [40]	Pneumatic conveying monitoring	Axial Flow	Passive (Relies on random diffusion)	400 to 6000 g/m^3^	Not reported	Not reported
Xu et al., 2021 [41]	Wind-sand transport monitoring	Axial Flow	Passive (Relies on natural wind impaction)	~29 g/m^3^	0.168 nA/(g·m^−3^)	Not reported
Our work	Dilute-phase emission and conveying	Swirling Flow	Active (Centrifugal force drives particles to sensing wall)	~30 g/m^3^ (10 μm) ~1400 g/m^3^ (1000 μm)	2.89 nA/(g·m^−3^) (10 μm) 0.031 nA/(g·m^−3^) (1000 μm)	Max > 22 dB (Eliminates blind zones down to ~5 g/m^3^)

## Data Availability

The data presented in this study are available on request from the corresponding author.
